# Identification of five important genes to predict glioblastoma subtypes

**DOI:** 10.1093/noajnl/vdab144

**Published:** 2021-10-10

**Authors:** Yang Tang, Maleeha A Qazi, Kevin R Brown, Nicholas Mikolajewicz, Jason Moffat, Sheila K Singh, Paul D McNicholas

**Affiliations:** 1 Department of Mathematics and Statistics, McMaster University, Hamilton, Ontario, Canada; 2 Department of Biochemistry and Biomedical Sciences, McMaster University, Hamilton, Ontario, Canada; 3 Terrence Donnelly Centre for Cellular and Biomolecular Research, University of Toronto, Toronto, Ontario, Canada; 4 Department of Surgery, McMaster University, Hamilton, Ontario, Canada

**Keywords:** classification, gene signature, glioblastoma subtypes, statistical learning, XGBoost

## Abstract

**Background:**

Glioblastoma (GBM), the most common and aggressive primary brain tumour in adults, has been classified into three subtypes: classical, mesenchymal, and proneural. While the original classification relied on an 840 gene-set, further clarification on true GBM subtypes uses a 150-gene signature to accurately classify GBM into the three subtypes. We hypothesized whether a machine learning approach could be used to identify a smaller gene-set to accurately predict GBM subtype.

**Methods:**

Using a supervised machine learning approach, extreme gradient boosting (XGBoost), we developed a classifier to predict the three subtypes of glioblastoma (GBM): classical, mesenchymal, and proneural. We tested the classifier on in-house GBM tissue, cell lines, and xenograft samples to predict their subtype.

**Results:**

We identified the five most important genes for characterizing the three subtypes based on genes that often exhibited high Importance Scores in our XGBoost analyses. On average, this approach achieved 80.12% accuracy in predicting these three subtypes of GBM. Furthermore, we applied our five-gene classifier to successfully predict the subtype of GBM samples at our centre.

**Conclusion:**

Our 5-gene set classifier is the smallest classifier to date that can predict GBM subtypes with high accuracy, which could facilitate the future development of a five-gene subtype diagnostic biomarker for routine assays in GBM samples.

**Key Points**

- Novel application of a machine learning classifier to glioblastoma (GBM) subtyping data.
- Identified five genes that were most important for predicting GBM subtypes with >80% accuracy.

**Importance of the Study**

To date, GBM subtyping relies on gene signatures developed using RNA-sequencing expression of more than 150 genes. In this study, we applied a supervised machine learning approach to identify the most important genes for accurate subtyping of GBM samples using publicly available GBM RNA-sequencing datasets and validated it in our in-house GBM RNA-sequencing data. Using this approach, we identified five genes that could predict GBM subtypes with a classification accuracy of over 80%. This 5-gene set signature can be developed into a diagnostic assay for GBM classification.

Glioblastoma (GBM) is the most common and malignant primary tumor affecting the adult nervous system (WHO Grade IV).^1^ For newly diagnosed primary GBM, a multi-modal treatment approach is undertaken including surgery, radiation, and chemotherapy. Despite this aggressive treatment, almost all patients with GBM relapse 7–9 months post-diagnosis. The 2-year survival rate for GBM stands at an abysmal 16.9% with only 5.5% of patients surviving at 5-years and 2.9% at 10-years.^2^

Given the highly treatment-resistant nature of GBM, studies from The Cancer Genome Atlas (TCGA) and Parsons et al. first dissected the mutational landscape of GBM with the aim of discovering actionable mutations or predictive signatures.^3,4^ With over 200 GBM samples characterized through DNA copy number, gene expression, and DNA methylation profiling, TCGA was able to identify three critical signaling networks that harbor the most frequent mutations: Receptor tyrosine kinase (RTK) signaling, p53 signaling, and RB signaling. Although the mutational landscape of GBM highlighted multiple avenues of putative therapeutic targeting, outcomes of clinical trials have to date been negative. Gene expression profiling further elucidated the gene signatures associated with clinical outcomes and patient survival.^5–7^ These studies showed that histopathological GBM actually represented multiple molecular subtypes with extensive inter-tumoral heterogeneity.

Using consensus average linkage hierarchical clustering of almost 200 GBM gene expression profiles, Verhaak et al. identified four transcriptomic subtypes of GBM: proneural, neural, classical, and mesenchymal, which were then validated in a separate 260 GBM dataset.^8^ Further studies using RNA-sequencing by Wang et al. have identified that only pro-neural, classical, and mesenchymal subtypes represent the glioma-intrinsic subtypes, while the neural subtype could have been identified earlier due to higher contamination of normal neural tissue in the tumor sample.^9^ The distinct underlying biology of the GBM subtypes also suggests a possible role of targeted therapy based on signaling networks that specifically govern each GBM subtype. More recently, single-cell RNA-sequencing of GBM by Neftel and colleagues has elucidated the presence of heterogeneous cellular states that populate each GBM and based on recapitulation of normal neural signatures are defined into four cell states: neural-progenitor-like, oligodendrocyte-progenitor-like, astrocyte-like, and mesenchymal-like.^10^ Although these transcriptional states have unique underlying genetic alterations, these states remain plastic and change in response to stimuli from their microenvironment. While these recent advances pave the path for greater understanding of functional intra-tumoural heterogeneity present in GBM and its impact on disease progression and treatment outcomes, current limitations due to cost and throughput associated with these technologies restrict their clinical application.^11^

To date, GBM subtyping has relied on consensus average linkage hierarchical clustering of gene expression profiles to categorize GBM samples into the different subtypes, with a 150 gene-signature allowing for robust classification across different sample batches and gene expression methodologies. However, with the recent advances in machine learning and its application to cancer biology, new methodologies that accurately assign subtype identity to GBM samples with fewer genes need to be developed to further facilitate improved routine diagnostic tests. Therefore, developing methodology to accurately classify GBM subtypes with small number of genes holds strong clinical value and maybe employed as a part routine laboratory testing. In this study, we use a supervised machine learning technique (ie, extreme gradient boosting) to predict existing GBM subtypes as well as to achieve strong predictive accuracy, based on just a few genes, using the RNA-seq dataset and the subtype labeling given to these samples by Wang et al.^9^

## Materials and Methods

### The RNA-seq GBM Dataset from Wang et al. (2017) and the Subtypes

For the purpose of the study, the GBM RNA-seq dataset generated by Wang et al. available on the GlioVis website is considered.^9,12^ The dataset consists of 20,501 variables for 160 individuals. After removing samples containing unavailable subtype information and the genes exhibiting zero counts across all samples, the dimension of the dataset is 19,980 genes for 156 GBM samples. The subtype classes consist of three subtypes including classical, mesenchymal, and proneural.^9^ This data contains the cases for 59 classical, 51 mesenchymal, and 46 proneural subtypes.

### Extreme Gradient Boosting

The method of analysis used for this data is known as extreme gradient boosting, more commonly referred to as XGBoost.^13^ It is a widely used technique that is essentially a scalable implementation of gradient boosting machines.^14^ Boosting refers to an ensemble method that can create a strong classifier that is based on iteratively applying weaker classifiers. This iterative process gradually reduces the classification error, on the training data, while overfitting can be avoided via clever tuning. Cross-validation (CV) is commonly used for tuning.

A benefit of using gradient boosting is that after the boosted trees are constructed, we are able to retrieve Importance Scores for each gene. First, a score is calculated for a single decision tree by the amount that each gene split point improves the classification accuracy, and then the Importance Score for each gene is averaged across all of the decision trees. Initially, we start with 1,000 trees and the maximum depth of each tree is set to 50. To avoid overfitting, 80% of the genes are randomly selected per tree. The classifier finishes training if the accuracy does not improve for 50 rounds.

### Cross-validation Based Gene Selection

We used repeated 10-fold CV to assess the performance of our classifier. In one round of 10-fold CV, the data are randomly partitioned into 10 roughly equally sized subsamples. Of these 10 subsamples, nine take the role of a training set and are used to build a classifier while the remaining subsample is used as a validation set to assess classification performance. This process is then repeated so that each subsample acts once as the validation set and the classification accuracy for the validation sets are then averaged, that is, one complete run of 10-fold CV involves 10 rounds. After performing one run of 10-fold CV on the Wang et al. (2017) GBM RNA-seq dataset, we calculated the average classification accuracy for the validation sets and recorded the top 20 genes, that is, the 20 genes with the highest Importance Scores. We carried out a total of 100 runs of 10-fold CV and, thereby, obtained 100 sets of top 20 genes as well as 100 averaged CV accuracies for subtype classification.

### RNA-sequencing of Human GBM Samples

Human GBM brain tumors were obtained from consenting patients, as approved by the Hamilton Health Sciences/McMaster Health Sciences Research Ethics Board. RNA was extracted from human IDH-wildtype GBM patient tissue (*n* = 11; primary GBM *n* = 7, recurrent GBM *n* = 4), GBM cell lines (*n* = 13), and patient-derived xenografts (PDX; *n* = 33) for RNA sequencing (McMaster Samples). Following RNA extraction, stranded sequencing libraries were prepared using the Illumina TruSeq Stranded Total RNA LT Sample Prep kit. Samples were sequenced on an Illumina HiSeq 2500 using 2 × 100 base pair paired-end reads. Reads were trimmed to eliminate adaptor contamination using the Illumina bcl2fastq-conversion software (v2.20), and any reads shorter than 36bp after trimming were removed. All reads were mapped to the Gencode v25 transcript models and hg38 human genome sequence using the STAR short-read aligner (v.2.4.2a), including the command line flag “--quantMode GeneCounts” to produce gene-level read count files. All read count files were merged together into a matrix for further analysis. To adjust for differences in read depth, the total number of reads for each sample (ie, column) was determined, and the sum was used to divide the read count for each gene and multiplied by 1M to produce “counts per million (CPM) mapped reads”.

## Results

### Classification Accuracy and Important Genes

The averaged CV classification accuracy across the 100 runs is 80.12% with a standard deviation of 0.011. The minimum CV accuracy is 77.71% and the maximum CV accuracy is 82.96%. A list of the genes most frequently identified as having high Importance Scores is presented in Table 1. We identify NKAIN1, UBE2E2, F13A1, RNF149, and PLAUR as the top five most important genes for classifying GBM subtypes as they appeared in the top 20 gene list at least 80 times out of 100 runs.

**Table 1.** Most Frequently Identified Genes When Classifying Classical, Mesenchymal, and Proneural Subtypes

	Variable	Frequency
1	NKAIN1	100
2	UBE2E2	99
3	F13A1	92
4	RNF149	81
5	PLAUR	80
6	TNFAIP8	63
7	PIPOX	59
8	CTSC	58
9	SLC2A10	56
10	MTSS1	51
11	FAM57B	46
12	LRRC16A	40
13	GNA15	38
14	EGFR	35
15	FGFR3	31
16	PLCXD2	30
17	DYRK3	30
18	PAX6	28
19	HEPACAM	27
20	ITGA7	25

### Biological Relevance of the Identified Genes: NKAIN1, UBE2E2, F13A1, RNF149, and PLAUR

**Figure 1.**
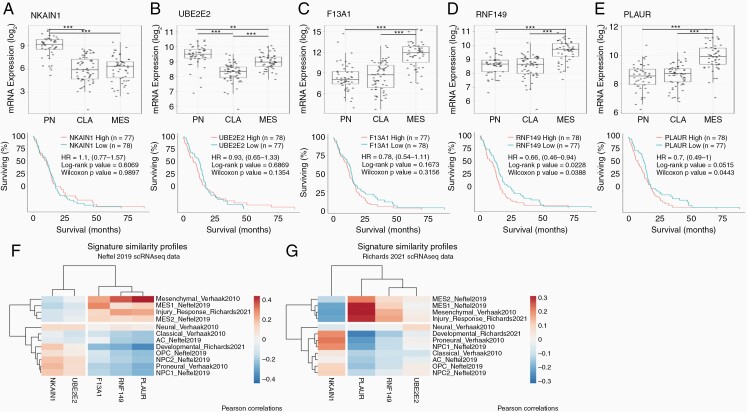
**Expression of 5-top genes across GBM subtypes and survival.** Top panels show the gene expression of the top-5 genes (A) NKAIN1, (B) UBE2E2, (C) F13A1, (D) RNF149, and (E) PLAUR across proneural (PN), classical (CLA), and mesenchymal (MES) subtypes of GBM in Wang et al. (2017) study. Bottom panel shows corresponding survival data of GBM samples with high or low expression of the top 5-genes. Signature similarity profiles of single-cell RNA sequencing from (F) Neftel et al. (2019)^10^ dataset and (G) Richards et al. (2021)^18^ dataset. HR = log Hazard Ratio. **P < .01, ***P < .001.

Knowing the strong predictive power of the top five genes, we were interested in investigating the biological relevance of these genes based on gene expression across subtypes and GBM survival analysis.^9,12^ Sodium/Potassium transporting ATPase Interacting 1 (NKAIN1) is a membrane-bound protein that interacts with beta subunit of the Sodium/Potassium ATPase.^15^ Although the gene is not well studied in GBM, NKAIN1 has the highest expression in proneural subtype as compared to both classical and mesenchymal subtype with no difference in survival (Figure 1A).

Ubiquitin conjugating enzyme E2 E2 (UBE2E2) is a cytoplasmic protein that plays a role in antigen processing and presentation for MHC complexes as well as a part of innate immunity. Proneural subtype has the highest expression of UBE2E2 as well, with mesenchymal subtype have a medium expression and classical subtype having the lowest expression (Figure 1B). High expression of EBE2E2 does not concur any changes in survival advantage for GBM patients.

Coagulation factor XIII A chain (F13A1) is a member of the blood coagulation cascade as a matrix cross-linker. Since extracellular matrix plays an important role in solid tumor growth, F13A1 may modulate aspects of the extracellular matrix to shape the tumor microenvironment in cooperation with tumor-associated macrophages.^16^ F13A1 has the highest expression in mesenchymal subtype of GBM, a subtype that is also associated with high infiltration of tumor-associated macrophages in the tumor microenvironment (Figure 1C).

Ring finger protein 149 (RNF149) functions as an E3 ubiquitin-protein ligase and may play roles in multiple signaling pathways, including proteosomal degradation and antigen processing. RNF149 is highly expressed in mesenchymal subtype of GBM as compared to classical and pro-neural subtypes (Figure 1D). High expression of RNF149 also predicts significantly poorer survival (log HR = 0.66[0.46–0.49], log-rank *P*-value = .0228), corresponding with the fact that high expression is correlated with poor-surviving mesenchymal subtype.

Plasminogen activating, urokinase receptor (PLAUR), a cell-membrane bound protein, plays a role in the localization and promotion of plasmin formation, which results in reduction or slowing down of clot formation. PLAUR also regulates and remodels the extracellular matrix and has been shown to promote *in vitro* GBM survival.^17^ The expression of PLAUR is the highest in the mesenchymal subtype of GBM and high expression also trends towards predicting poor survival in GBM patients (log HR = 0.7[0.49–1], log-rank *P*-value = .0515) (Figure 1E).

To assess whether these five genes also discriminated between GBM subtypes in other GBM datasets, we performed signature similarity analysis using GBM-specific gene sets in two independent single-cell RNA-sequencing GBM datasets (Neftel^10^ and Richards^18^ datasets) and evaluated the correlation between their signature scores and expression of the five genes (Figure 1F and G). Similar to differential expression of these genes across the three GBM subtypes in Wang et al. dataset (Figure 1A–E), we found that NKAIN1 and UBE2E2 correlated with proneural/classical-like subtypes while F13A1, RNF149, and PLAUR correlated with mesenchymal-like subtypes in GBM.

## Application

### Prediction of McMaster Samples' Subtypes Using Top Five Genes

We tested the classifier in a new GBM RNA-seq dataset (*n* = 57 McMaster cohort; described in Methodology 2.4), which consisted of primary and recurrent human GBM tissue samples, their corresponding *in vitro* generated cell lines, and *in vivo* xenografted primary GBM samples that were subjected to chemoradiotherapy at sequential time points.

The five genes we identified with strong predictive power were used to build 100 classifiers. Then one set of predictions for the 57 samples as well as the averaged CV accuracy was recorded for each classifier using the Wang et al. RNA-seq GBM data. The average CV classification accuracy across the 100 runs with these five genes is 83.28% with a standard deviation of 0.013. The CV accuracy with the Wang et al. RNA-seq data is expected to be higher in this case because these five genes are chosen on the basis of all the Wang et al. GBM samples. We assign a subtype to a case when the count of the subtype is more than 80 out 100 times. Table 2 shows the detailed subtype prediction for the 100 runs using the McMaster cohort. For most of the samples, the predictions are consistent across all classifiers.

**Table 2.** Detailed Subtype Prediction for the 100 Runs Using McMaster Sample Cohort. PDX: Patient-derived Xenograft

Patient ID	Sample Type	Classical	Mesenchymal	Proneural	Subtype Prediction
BT241	Cell Line	1	99	0	Mesenchymal
BT241	PDX	1	99	0	Mesenchymal
BT428	Cell Line	1	1	98	Proneural
BT428	PDX	1	1	98	Proneural
BT428	PDX^a^	1	38	61	
BT428	PDX	1	1	98	Proneural
BT428	PDX	0	1	99	Proneural
BT428	PDX^a^	0	0	100	Proneural
BT594	Cell Line	100	0	0	Classical
BT618	Cell Line	0	99	1	Mesenchymal
BT618	PDX	0	99	1	Mesenchymal
BT618	Patient Tissue	0	100	0	Mesenchymal
BT667	Cell Line	0	99	1	Mesenchymal
BT667	PDX	0	0	100	Proneural
BT667	PDX	0	99	1	Mesenchymal
BT667	PDX^a^	15	85	0	Mesenchymal
BT667	Patient Tissue	0	100	0	Mesenchymal
BT667	Patient Tissue	0	100	0	Mesenchymal
BT667	PDX	11	89	0	Mesenchymal
BT667	PDX^a^	26	73	1	
BT698	Cell Line	1	61	38	
BT698	Patient Tissue	0	23	77	
BT799	Cell Line	0	0	100	Proneural
BT799	PDX	0	0	100	Proneural
BT799	PDX^a^	0	0	100	Proneural
BT799	PDX	0	0	100	Proneural
BT799	Patient Tissue	0	0	100	Proneural
BT935	Cell Line	3	1	96	Proneural
BT935	PDX	74	0	26	
BT935	PDX	87	0	13	Classical
BT935	PDX^a^	0	0	100	Proneural
BT935	Patient Tissue	0	100	0	Mesenchymal
BT935	PDX	3	2	95	Proneural
BT935	PDX^a^	7	0	93	Proneural
BT954	Cell Line	79	21	0	
BT954	PDX	80	5	15	Classical
BT954	PDX	80	5	15	Classical
BT954	PDX^a^	80	5	15	Classical
BT954	Patient Tissue	0	40	60	
BT954	PDX	80	5	15	Classical
BT954	PDX^a^	80	5	15	Classical
BT956	Cell Line	1	99	0	Mesenchymal
BT956	Patient Tissue	0	99	1	Mesenchymal
BT972	Cell Line	94	6	0	Classical
BT972	PDX	99	1	0	Classical
BT972	Patient Tissue	0	80	20	Mesenchymal
MBT06	Cell Line	0	100	0	Mesenchymal
MBT06	PDX	13	87	0	Mesenchymal
MBT06	PDX	1	99	0	Mesenchymal
MBT06	PDX^a^	0	2	98	Proneural
MBT06	PDX^a^	0	28	72	
MBT06	PDX	0	2	98	Proneural
MBT06	PDX^a^	1	97	2	Mesenchymal
MBT06	PDX^a^	2	95	3	Mesenchymal
MBT27	Cell Line	2	0	98	Proneural
MBT27	Patient Tissue	0	100	0	Mesenchymal
MBT96	Patient Tissue	0	94	6	Mesenchymal

^a^PDX models treated with combination of radiation, chemotherapy, and/or targeted therapy.

### Comments on the Prediction

Identification of GBM subtypes allowed for a greater understanding of the underlying biology that governs GBM inter-tumoral heterogeneity. To date, consensus average linkage hierarchical clustering—an unsupervised learning technique—has been used to analyze GBM transcriptomic data and determine GBM subtype. By applying the supervised machine learning technique XGBoost to available GBM subtyping data, we identified five genes that can predict GBM subtype with high accuracy. These genes, although studied within the context of GBM biology, have not been previously described as important candidates for subtype identification of GBM samples. By applying our 5-gene classifier, we are able to accurately predict the subtype of new GBM samples from patient tissue samples as well as *in vitro* and *in vivo* studies, as demonstrated through the McMaster samples. While the majority of samples could be classified to a single subtype based on our cutoff of at least 80 out of 100 runs, some samples from all three sample types from McMaster cohort could not be classified to a single GBM subtype based on this criteria (Table 2; patient tissue *n* = 2/11, cell line *n* = 2/13, PDX *n* = 4/33). Although patient tissue samples were more likely to not be classified into a single subtype as compared to cell lines and PDX samples, which may be suggestive of higher prevalence of intra-tumoral heterogeneity in tissue samples, overall our classifier predicted subtypes for over 80% of the samples in each sample type. Interestingly, the same two patient tissue samples that could not be classified also did not have their corresponding cell lines classified (BT698 and BT954, Table 2), which maybe suggestive of higher intratumoral heterogeneity of these samples that limit clear subtype classification. Moreover, in some instances, the same GBM patient sample switched subtypes between tissue cell lines and PDX, signifying that the transcriptional programs underlying GBM subtypes change in response to environmental stimuli such as cell culture conditions, PDX microenvironment, and treatments (radiation, chemotherapy, and/or targeted therapy in PDX models). Further studies on the biological basis of the five genes in GBM subtype-prediction can lead to greater understanding of how GBM subtypes develop in the context of tumor progression.

## Conclusion

In this study, we built a classifier to predict the three subtypes of GBM: classical, mesenchymal, and proneural. Our approach achieved 80.12% accuracy on average in predicting these three subtypes of GBM. We identified the five most important genes for characterizing the three subtypes based on genes that often had high Importance Scores in our XGBoost analyses. We applied our five-gene classifier to successfully predict the subtype of GBM samples at our centre (McMaster cohort). Given that our classifier consists of a small number of genes, future studies need to be undertaken to develop and evaluate the utility of a five-gene subtype diagnostic subtype biomarker through the use of cost-effective technologies such as NanoString assays or evaluated as part of RT-PCR or immunohistochemistry panels that are routinely assayed in all GBM patients.

## Data Availability

The data used to develop the 5-gene classifier is publicly available at http://gliovis.bioinfo.cnio.es/.

## Code Availability

The XGBoost python module was used and is publicly available.
